# Probabilistic inversion of expert assessments to inform projections about Antarctic ice sheet responses

**DOI:** 10.1371/journal.pone.0190115

**Published:** 2017-12-29

**Authors:** Robert William Fuller, Tony E. Wong, Klaus Keller

**Affiliations:** 1 Department of Geosciences, The Pennsylvania State University, University Park, Pennsylvania, United States of America; 2 Earth and Environmental Systems Institute, The Pennsylvania State University, University Park, Pennsylvania, United States of America; 3 Department of Engineering and Public Policy, Carnegie Mellon University, Pittsburgh, Pennsylvania, United States of America; Southwest University, CHINA

## Abstract

The response of the Antarctic ice sheet (AIS) to changing global temperatures is a key component of sea-level projections. Current projections of the AIS contribution to sea-level changes are deeply uncertain. This deep uncertainty stems, in part, from (i) the inability of current models to fully resolve key processes and scales, (ii) the relatively sparse available data, and (iii) divergent expert assessments. One promising approach to characterizing the deep uncertainty stemming from divergent expert assessments is to combine expert assessments, observations, and simple models by coupling probabilistic inversion and Bayesian inversion. Here, we present a proof-of-concept study that uses probabilistic inversion to fuse a simple AIS model and diverse expert assessments. We demonstrate the ability of probabilistic inversion to infer joint prior probability distributions of model parameters that are consistent with expert assessments. We then confront these inferred expert priors with instrumental and paleoclimatic observational data in a Bayesian inversion. These additional constraints yield tighter hindcasts and projections. We use this approach to quantify how the deep uncertainty surrounding expert assessments affects the joint probability distributions of model parameters and future projections.

## Introduction

Sea-level rise increases risks to coastal communities [1]. Approaches to managing these risks include augmenting levees, adding new flood control and shoreline protection structures, diverting sediments, improving infrastructure, relocating vulnerable populations, and restoring or enhancing natural coastal protections, such as barrier reefs, barrier islands, ridges, marshes, and regional hydrology [2,3]. The design of sound risk management strategies depends on quantifying and characterizing the uncertainties surrounding sea-level projections [4,5]. Projections of future sea-level rise depend on deeply uncertain projections of Antarctic ice sheet (AIS) mass loss [4,6–9].

Deep uncertainty arises, for example, when experts and/or decision-makers disagree about model structure or prior probabilities of key model parameters [10]. Some sources of AIS deep uncertainty include (i) the difficulties in representing recently discovered mechanisms affecting AIS response, such as marine ice shelf instability (MISI) and marine ice cliff instability (MICI) [11,12], and (ii) the difficulties in calibrating these models with observations [7,13]. As a result, projections of the AIS contribution to global sea level are often characterized by divergent expert assessments [5,9]. Probabilistic inversion may provide a way to represent and quantify this deep uncertainty. Probabilistic inversion can fuse expert assessments with mechanistically-motivated models to infer expert prior distributions for model parameters and to sample the uncertainty due to divergent (interpretations of) expert assessments [6].

The scarcity of AIS instrumental and paleoclimatic observations limits the ability to constrain key model parameters [4,6,13]. This elevates the importance of the prior distributions assumed for the model parameters. The complexity of the processes that determine the AIS dynamical response can lead to high-dimensional models with many correlated model parameters. Eliciting prior distributions for these high dimensional probability density functions—about which there is often little intuition—poses nontrivial challenges (e.g., [5,14]). Model parameters lacking clear physical meaning compound this problem [15]. Probabilistic inversion provides a means to infer these prior model parameters from expert assessments of future observations [16,17], such as AIS mass loss by the year 2100 [18].

Probabilistic inversion of expert assessments first appeared prominently in peer-reviewed literature in 2000 in the context of mitigating nuclear risks [19]. In the context of sea-level rise, an early use of a probabilistic inversion technique, via rejection sampling, appeared about a decade later [20]. More recently, probabilistic inversion was used in the context of climate change [6]. Typically, probabilistic inversion employs the iterative proportional fitting (IPF) algorithm [6,16,17]. Markov chain Monte Carlo (MCMC) integration of intractable model parameter probability distributions offers an alternative to IPF for high-dimensional models [21,22].

Advantages to using MCMC for probabilistic inversion include (i) good resolution of the tails of probability distributions [23,24] which can be critical from a risk-management perspective (e.g., [6,7,20]), (ii) theoretical convergence diagnostics [25–27], and (iii) the ability to subsample or post-process Markov chains [28]. On the other hand, MCMC can be computationally expensive. The computational demands for this study are relatively low, however; producing a five million member Markov chain requires roughly 30 hours of computer processing time. This study requires eight such chains which we produce in parallel on a high-performance computing cluster.

Here, we employ probabilistic inversion [16,17] to fuse different interpretations of an expert assessment [18] with a simple AIS model, modified to include a rudimentary mechanism for the potential rapid AIS ice loss [7,13,29]. We demonstrate that probabilistic inversion leads to expert prior model parameters that are consistent with the expert assessments. Then, we couple probabilistic inversion and Bayesian inversion. This coupled probabilistic-Bayesian inversion combines expert assessments with paleoclimatic and instrumental AIS observational data. The resulting posterior parameter estimates and sea-level projections exhibit tighter constraints relative to the expert prior model parameters and projections, showcasing the value of combining the two information streams. We demonstrate the impact of varying interpretations of expert assessments on model projections of Antarctic ice sheet mass loss by 2100. Finally, we characterize deep uncertainty by quantifying the impacts of these divergent expert assessments on prior model parameters and projections. We constrain estimates of all model parameters using this method (see Supporting Information, S5–S7 Figs). However, we focus the analysis on the two parameters relevant to the potential fast Antarctic ice sheet dynamics, as this mechanism is a key potentially decision-relevant deep uncertainty [4,7].

## Methods

### Model

We employ the Danish Center for Earth System Science (DCESS) Antarctic ice sheet (DAIS) model [13,29], modified to capture AIS fast dynamical contributions to sea-level rise [7]. DAIS simulates an idealized ice sheet that is symmetric around a central vertical axis. The model averages processes around the ice sheet rather than distinguishing between individual basins, and follows a mass balance formulation that accounts for precipitation, runoff from melt, and ice flow into the ocean from the ice sheet periphery. Ice flow is calculated as (i) proportional to the radius of the ice sheet, (ii) proportional to the height of the ice at the periphery, which captures processes such as marine ice shelf instability (MISI) [13], and (iii) exponentially proportional to the difference between Antarctic ocean subsurface temperature and sea-level temperature, which captures processes such as basal melt [29]. The DAIS model contains many of the key ice sheet physical processes, while approximating some of the relevant mechanisms, making it computationally efficient enough to enable probabilistic inversion and calibration [13].

The addition of an explicit model representation of fast AIS dynamical disintegration processes [7] enables DAIS to approximate recently discovered AIS physical processes, such as hydro-fracturing and marine ice cliff instability (MICI) [11,12]. In order to keep things simple, fast dynamical contributions are included directly in the mass balance rather than indirectly through the ice flow at the periphery [7]. Fast dynamical disintegration is triggered when the Antarctic sea-level temperature rises above *T*_*crit*_ (°C) and the volume of the ice sheet is greater than 18 million km^3^, which we approximate as the volume of ice susceptible to fast dynamics [7,30]. Fast dynamical ice sheet disintegration is assumed to occur at rate *λ* (mm yr^-1^). This parameterization follows *Diaz and Keller* [31] and *Wong et al*. [7]. As noted by *Wong et al*. [7], the relationship between *T*_*crit*_ and fast AIS disintegration is not a causal one, but rather *T*_*crit*_ serves as an indicator of other relevant mechanisms leading to fast disintegration. We use a Markov chain Monte Carlo method to jointly sample the uncertainty in *T*_*crit*_ and *λ* [7,32], as well as 13 other model parameters [13]. Sampling *T*_*crit*_ produces a probabilistic estimate of the temperature that is associated with accelerated disintegration of the AIS. Thus, this coupled physical-statistical model enables learning about key components of the physical system represented by this simple, yet informative, model.

### Expert assessments

We adopt published expert assessments to sample some of the deep uncertainty (i.e. multiple probabilistic assessments) surrounding the projections of AIS dynamics. Specifically, the Low 2 and High 1 projections for Antarctica from Table 3 in *Pfeffer et al*. [18] imply a probable range of 128 mm to 619 mm of sea-level rise from the AIS by the year 2100, relative to the year 2010. The study does not specify a probability distribution or how to interpret the range. Here, we adopt three mathematical forms that approximate past interpretations: (i) a uniform distribution (as an imputation to *Pfeffer et al*. [18]), which treats all levels of AIS sea-level rise as equally probable within the given range, (ii) a normal distribution (approximating *Church et al*. [33]), derived from taking each bound of the 128–619 mm range as representing plus and minus two standard deviations from the mean of the range, giving a mean of 373.5 mm and a standard deviation of 122.75 mm, and (iii) a beta distribution (e.g., [20,34]) with the lower bound equal to 128 mm, the upper bound equal to 619 mm, the shape parameter α = 2, and the shape parameter β = 3. This allows us to quantify the impact of different assumptions about the distribution of the expert assessments.

### Observations and constraints

We select observations for the full calibration from previous work using the DAIS model [7,13]. We incorporate AIS paleoclimatic data, instrumental data, and modelled trends. All AIS mass balance data is specified in global mean sea-level equivalents (SLE), relative to the mean of the 1961–1990 period, unless otherwise stated or implied.

Paleoclimatic data includes the Last Interglacial (LIG, about 118 kyr BCE), the Last Glacial Maximum (LGM, about 18 kyr BCE), and the Mid-Holocene (MH, about 4 kyr BCE). We select the LIG constraint of 3.6 m to 7.4 m SLE from *DeConto and Pollard* [11]. We treat the LIG as normally distributed with a mean of 5.5 m and a standard deviation of 0.95 m, and truncate the distribution at two standard deviations above and below the mean. We choose the normally-distributed LGM and MH constraints from *Ruckert et al*. [13] with means of -11.35 m and -2.63 m and standard deviations of 2.23 m and 0.69 m, respectively.

The instrumental data includes estimated AIS mass loss and global mean sea-level data. We adopt *Shepherd et al*. [35] for mass loss during the instrumental period. For instrumental year 2002, we take AIS mass loss to be normally-distributed with a mean of 1.97×10^−3^ m SLE and a standard deviation of 4.7×10^−4^ m per *Ruckert et al*. [13]. Following *Wong et al*. [7], we incorporate a Heaviside function to disallow model simulations in which AIS mass loss (in SLE) exceeds global mean sea-level rise [36] for each of the instrumental years from 1900 to 2013 CE.

Lastly, we constrain the rate of AIS mass loss for multiple recent periods with information from the IPCC assessment [33]. Specifically, we consider rates of AIS mass loss for the years 1993 to 2010 CE, 1992 to 2001 CE, and 2002 to 2011 CE [33]. These have means of 0.27 mm y^-1^ SLE, 0.08 mm y^-1^, and 0.40 mm y^-1^ respectively with corresponding standard deviations of 0.11 mm y^-1^, 0.185 mm y^-1^, and 0.205 mm y^-1^.

### Climatic forcings

Global mean sea level, Antarctic sea-level temperatures, and Antarctic ocean subsurface temperatures force the DAIS model. We adopt climatic forcings following *Ruckert et al*. 2017 [13]. For the period from 238 kyr BCE to the year 1997 CE, we use climatic forcings from *Shaffer* [29]. For the years 1997 to 2100 CE, we use forcings as generated for *Ruckert et al*. [13] from the extended Representative Concentration Pathways (RCP) 8.5 scenario [37].

### Statistical calibrations

**Fig 1:**
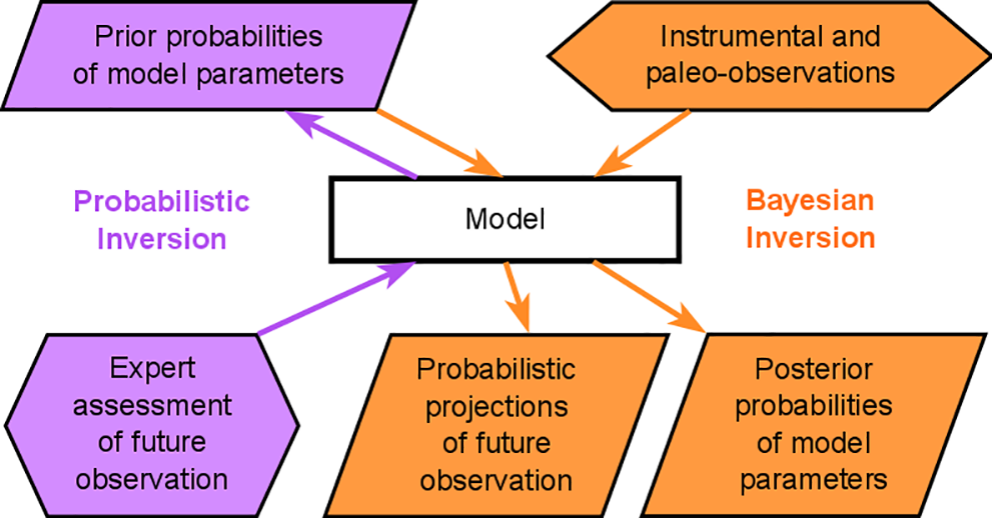
Schematic of coupled probabilistic-Bayesian inversion. Hexagons indicate observational constraints. Parallelograms represent probability distributions: prior, posterior, and predictive. Rectangle denotes physical and statistical model. Arrows contrast the direction of inference in the coupled inversions.

We perform two interacting inversions: (i) probabilistic inversion of expert assessments and (ii) Bayesian inversion of instrumental and paleoclimatic observations (Fig 1). First, we use probabilistic inversion to fuse the DAIS model with each of the three interpretations of the expert assessments (uniform, normal, and beta distributions). By inverting the expert assessments with the DAIS model, we inform the prior probabilities of model parameters (Fig 1). Next, we add instrumental [35,36] and paleoclimatic constraints [11,13], as well as AIS mass loss trends [33], to each of the three expert assessments in a coupled probabilistic-Bayesian inversion. This results in six statistical calibration experiments: three probabilistic inversions and three coupled probabilistic-Bayesian (hybrid) inversions.

We implement the inversions using MCMC, by employing an adaptive Metropolis-Hastings algorithm [32]. The Metropolis-Hastings algorithm [23,24] samples from a target posterior probability distribution, given a likelihood function and prior probability distribution, that together are proportional to the target probability distribution (by Bayes’ theorem), conditioned on the data employed. The likelihood function evaluates the probability of each data constraint according to its assumed probability distribution and the model output for a given set of model parameters. For the three probabilistic inversions, the likelihood function calculates the conditional probability of the expert assessments as a function of the model parameters. For the three hybrid inversions, our likelihood function provides the conditional probability of the expert assessments, the paleoclimatic and instrumental constraints, and the IPCC modelled trends, as a function of the model parameters.

We adopt wide, uncorrelated prior model parameter distributions from *Wong et al*. [7]. We employ gamma prior distributions for the fast dynamics parameters, *T*_*crit*_ and *λ* from *Wong et al*. [7], as well as an inverse gamma prior for a variance parameter and uniform prior distributions for the remaining 12 DAIS model parameters, following previous work [13]. The probabilistic inversion experiments include only the expert assessments in the likelihood function. We use these experiments to evaluate the extent to which the expert assessments update the wide prior probability distributions of the model parameters [15]. For the hybrid inversions, we infer posterior probabilities for the model parameters from all of the constraints, including the expert assessments and observations, and the assumed wide prior probabilities of the model parameters.

We follow *Wong et al*. [7] to calibrate the hybrid inversions, with a few changes. First, we employ two separate statistical parameters to represent the variance for the paleoclimatic and instrumental observations as in *Ruckert et al*. [13]. Second, we only consider the RCP8.5 scenario for future projections. Third, we include the expert assessments in the likelihood function.

We produce Markov chains from 5×10^6^ iterations of the Metropolis-Hastings algorithm for each of the six inversions. We discard the first 250,000 iterations of each chain for burn-in. We use Gelman and Rubin’s potential scale reduction factors to diagnose convergence [27].

We post-process the Markov chain for the uniform probabilistic inversion using rejection sampling [28] to improve the fit to the uniform expert assessment. Others have experienced challenges in inverting uniform distributions as well (e.g., [15,38]). The normal and beta probabilistic inversions did not require rejection sampling. The random-walk Metropolis-Hastings algorithm seems adequately guided by the probability differing throughout the range of the normal and beta expert assessments.

## Results

### Probabilistic inversion

**Fig 2:**
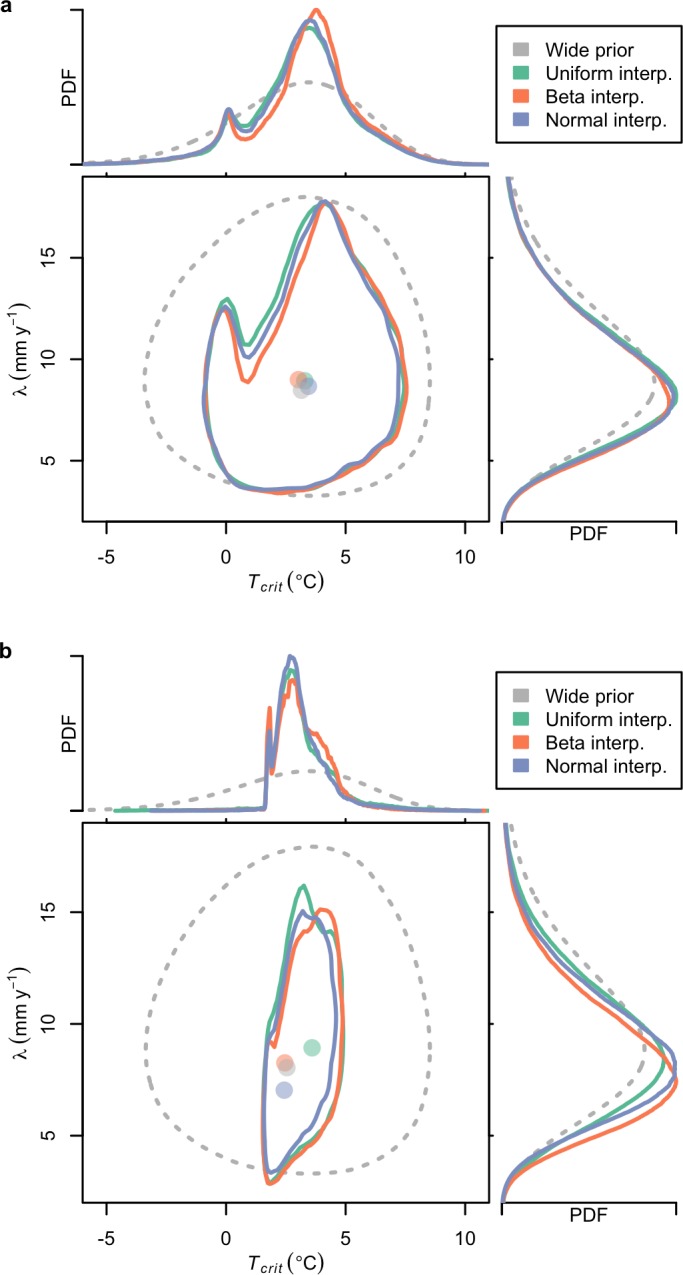
Inferred probability density functions. Shown are marginal posterior distributions of fast dynamics parameters from the (a) probabilistic inversion of the expert prior and the (b) combination of the expert prior with the observations using coupled probabilistic-Bayesian inversion. Contours delimit the 90% credible interval. Shaded circles indicate the mode. Temperature is scaled to global mean surface temperature.

**Fig 3:**
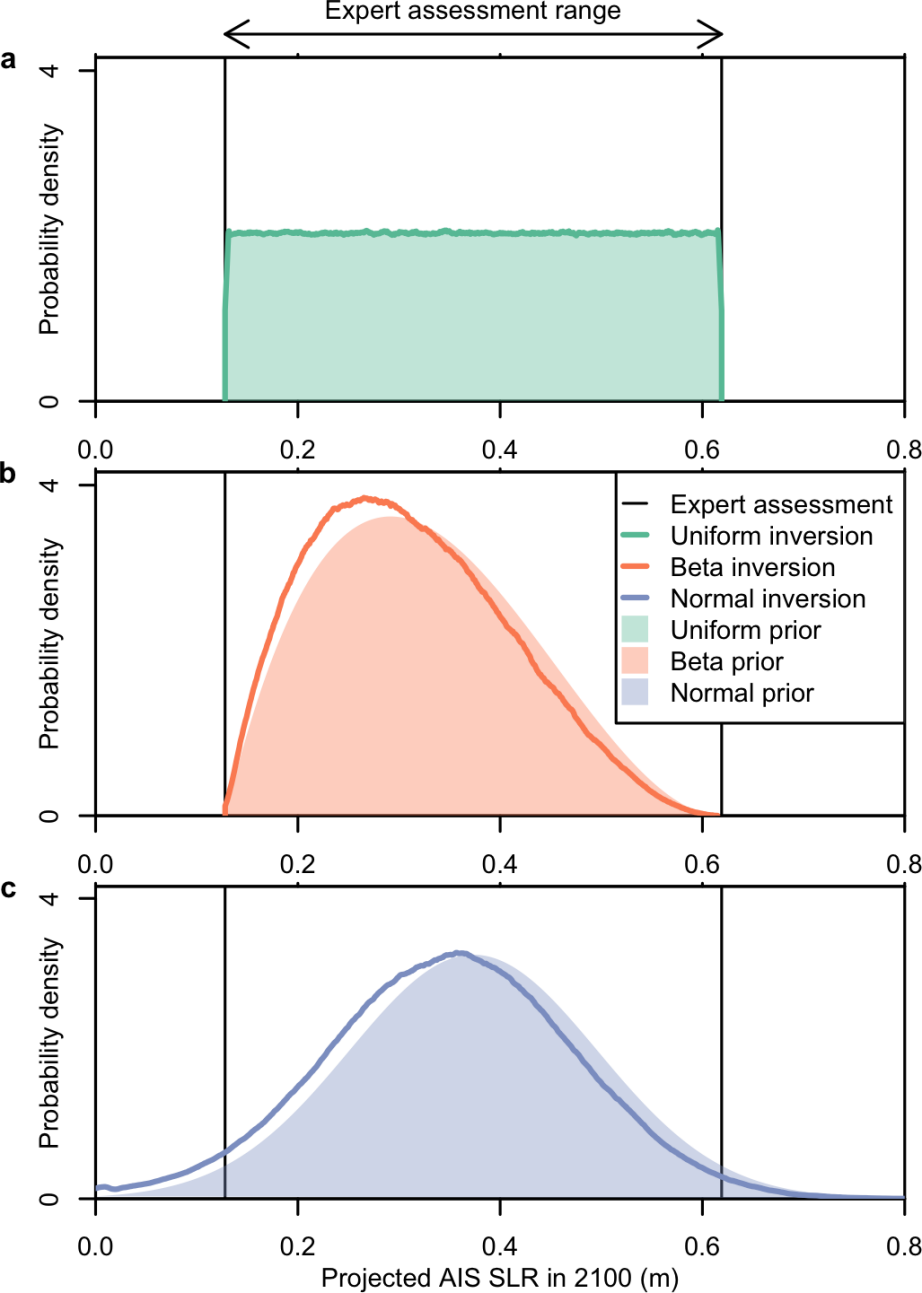
Probabilistic inversion of different interpretations of expert assessments. Shaded areas denote expert prior probability distribution. Lines give posterior expert probability distribution from probabilistic inversion. Shown are (a) uniform, (b) beta, and (c) normal interpretations. Vertical lines demarcate the range provided by *Pfeffer et al*. [18].

The probabilistic inversions produce weakly multimodal expert priors for the fast dynamics parameters, *T*_*crit*_ and *λ* (Fig 2A). (See also the other DAIS model parameters’ probability distributions for the inversions, S5–S7 Figs). The inferred expert priors generate model simulations that are readily able to approximately reproduce the three different interpretations of the expert assessment (Fig 3). We do not expect the approximations to be exact because we are using the expert assessments as conditional distributions to update the assumed wide priors [15].

### Inferred fast dynamics priors

The expert prior distributions inferred for the fast dynamics parameters show a secondary mode in their marginal probability distribution (Fig 2A). Adding paleoclimatic and instrumental observations to the information from the expert assessments sharpens the inference of the fast dynamics parameters (Fig 2B and Table 1). The Last Interglacial constraint largely eliminates the secondary mode (Fig 2). We discuss this further in the Supporting Information (S1 Text and S3 and S4 Figs). (See also the hindcast of the Last Interglacial and other additional constraints, S8 Fig).

**Table 1:** Quantiles for parameters and projections.

		*T*_*crit*_ (°C), GMST			SLE by year 2100 (m)		
Evidence	Expert Interp.	5%	mean	95%	5%	mean	95%
Wide priors	n/a	-2.00	2.9	7.1	n/a	n/a	n/a
+instrument+paleo+IPCC		1.50	3.1	7.0	0.03 m	0.29 m	0.75 m
Wide priors +expert Assessment	uniform	-0.40	3.3	6.6	0.15 m	0.37 m	0.59 m
	beta	-0.59	3.6	6.8	0.17 m	0.30 m	0.48 m
	normal	-0.60	3.3	6.7	0.14 m	0.35 m	0.55 m
Wide priors+expert +instrumental +paleo+IPCC	uniform	1.8	2.9	5.6	0.15 m	0.37 m	0.58 m
	beta	1.8	3.0	5.0	0.18 m	0.33 m	0.49 m
	normal	1.9	2.9	5.2	0.15 m	0.38 m	0.55 m

The estimate of the temperature associated with AIS fast disintegration, *T*_*crit*_, is sharpened by including the expert assessments in the coupled probabilistic-Bayesian inversion (Table 1). The Bayesian inversion without the expert assessments gives a 90% credible interval of 1.5–7.0°C, scaled to global mean surface temperature [39] from Antarctic sea-level temperature [29]. We narrow this range to 1.8–5.6°C by incorporating the uniform expert assessments into the coupled probabilistic-Bayesian inversion. The Bayesian inversion without the expert assessment tightens up the 5% quantile of the inferred distribution for *T*_*crit*_—from a prior of -2.00°C to a posterior of 1.5°C—whereas probabilistic inversion of the uniform expert assessment without the Bayesian inversion tightens up the 95% quantile for *T*_*crit*_—from a prior of 7.1°C to an inferred prior of 6.6°C. We find probabilistic inversion and Bayesian inversion to be complementary means of estimating key model parameter uncertainty, particularly in the case of *T*_*crit*_. However, the addition of the observational data does little to further constrain the disintegration rate, *λ*. This indicates that the assimilation of additional data streams may be needed.

### Posterior projections

**Fig 4:**
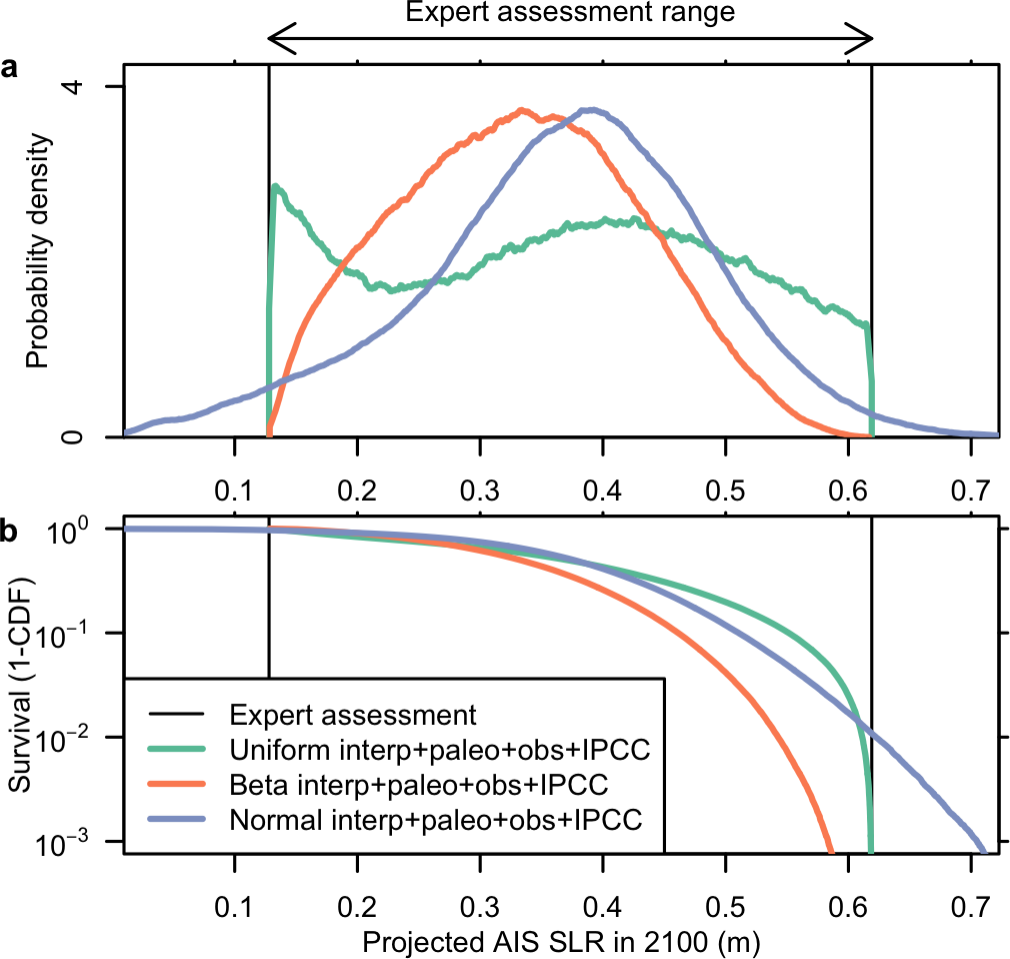
Posterior probability density functions. Sea-level projections inferred by combining expert assessments, paleoclimatic data, instrumental observations, and IPCC information [33]. Shown are (a) uniform, (b) beta, and (c) normal interpretations of the expert assessments. Vertical lines demarcate the range provided by *Pfeffer et al*. [18].

We find that the projected AIS contribution to sea-level rise hinges considerably on the interpretation of the expert assessments, particularly in the tail areas of the probability distributions. This is illustrated by the survival functions (Fig 4). As expected, the probabilistic inversion tightly constrains the projected AIS contribution to sea level by 2100, and these projections are relatively unchanged by including the observational data in the coupled probabilistic-Bayesian inversion. For example, the 90% credible interval for the probabilistic inversion of the normal interpretation of the expert assessments is 0.14–0.55 m SLE from the Antarctic ice sheet by the year 2100 (Table 1). Adding the paleoclimatic and instrumental constraints tightens these projections only slightly, with a 90% credible interval of 0.15–0.55 m SLE. We note that while the projections are relatively unchanged by the addition of the observational constraints, these data improve posterior inference regarding key model parameters (Fig 2). This suggests that the expert assessments are consistent with the observational constraints and model.

## Discussion and caveats

We intend this paper to be viewed pedagogically rather than as an exhaustive treatment of the problem domain. Specifically, this study illustrates a method for combining expert assessments with paleoclimatic and instrumental observations in a coupled probabilistic-Bayesian inversion. We quantify the effects of combining expert assessments with observational data in a model inversion. We find that the two techniques can be integrated to constrain future projections as well as key model parameters. This coupled-probabilistic Bayesian inversion technique can also be more widely applied to other problem domains.

For the sake of simplicity, we examine several interpretations of one expert assessment [18]. Future work might further characterize the deep uncertainty surrounding future AIS contributions to sea-level rise by considering multiple expert assessments and/or more complex model structures. Here we apply probabilistic inversion to one expert’s assessments whereas algorithms such as IPF have been used to invert and reconcile multiple experts’ assessments. IPF also lends itself to inverting multi-variate distributions of expert assessments. For example, total sea-level rise could be inverted from multiple experts’ assessments of the constituent components of sea-level rise, such as thermosteric expansion, the Greenland ice sheet, the Antarctic ice sheet, and other glaciers [17].

Another promising avenue would be to combine multiple expert assessments into a possibility function (see for example, [9]), then use the methods presented here to invert a possibility function along with paleoclimatic and observational data. The possibility function could include higher estimates for AIS contributions from more recent studies such as *DeConto and Pollard* [11], for example, but this is beyond the scope of this study. Also, IPF could be used to reconcile multiple divergent expert assessments, although feasibility may be an issue [6].

Future calibrations could include additional data. For example, we only consider the high-emissions RCP8.5 scenario here [37], whereas others include more scenarios (see for example, [7]). One could also calibrate the model with paleoclimatic data extending back to the Pliocene—between 5.3 and 2.6 million years ago—during which more AIS fast dynamical behavior occurred [11]. Thus, including paleoclimatic data from the Pliocene should further constrain the fast dynamics parameters.

Many of the caveats discussed elsewhere apply to this study as well (e.g., [7,13]). Briefly, the DAIS model emulates fast disintegration of the Antarctic ice sheet with a single threshold temperature, *T*_*crit*_, and a single rate of disintegration, *λ*. A more detailed physical model could explicitly resolve the individual processes, such as hydro-fracturing, marine ice shelf instability (MISI), and marine ice cliff instability (MICI). It seems unlikely that these processes all share a single response timescale or triggering mechanism. Moreover, a threshold response might exhibit hysteresis. In other words, if the temperature were to fall below the trigger temperature, *T*_*crit*_, ice sheet disintegration might still continue. Nevertheless, we show the utility in combining probabilistic inversion and Bayesian inversion, and use this coupled inversion technique to characterize the deep uncertainty in future Antarctic ice sheet contributions to sea level.

## Conclusions

We demonstrate how a coupled probabilistic-Bayesian inversion may be used to combine expert assessments with paleoclimatic and instrumental data in order to make probabilistic projections of future sea-level rise from the Antarctic ice sheet. We use probabilistic inversion to illustrate how inverting expert assessments of future mass loss with a simple Antarctic ice sheet model can be used to inform the prior probabilities of key model parameters. We show that combining expert assessments and observations in a coupled probabilistic-Bayesian inversion sharpens the inference of model parameters. Adding the expert assessments to the observations narrows the 90% credible interval for *T*_*crit*_, the global mean warming associated with Antarctic ice sheet fast disintegration, from 1.5–7.0°C to 1.8–5.6°C (Table 1). While projections of Antarctic ice sheet contributions to sea levels remain deeply uncertain, this work provides guidance on how we can leverage the available data to constrain these projections as well as key model parameters.

## Supporting information

S1 Fig Comparison of different interpretations of expert assessments. Lines give posterior probability distribution from probabilistic inversion of expert assessments. Vertical lines demarcate the range provided by *Pfeffer et al*. [18].
(TIF)

S2 Fig Expert assessments vs. all data. Dashed lines give sea-level estimates from probabilistic inversion of expert assessments. Solid lines give sea-level estimates from combining expert assessments, paleoclimatic data, instrumental observations, and modelled trends. Shown are (a-b) uniform, (c-d) beta, and (e-f) normal interpretations of the expert assessments. Vertical lines demarcate the range provided by *Pfeffer et al*. [18].
(TIF)

S3 Fig Probabilistic inversion sampling of fast dynamics parameters. Shown are (a) covariance of *T*_*crit*_ and sea-level estimates, (b) covariance of *λ* and sea-level estimates, and (c) marginal posterior distribution of fast dynamics parameters from probabilistic inversion of the uniform expert prior with all other parameters fixed at their joint maximum likelihood estimate.
(TIF)

S4 Fig Latin hypercube sampling of fast dynamics parameters with widened priors. Shown are (a) all model runs and the (b) subset of those model runs that fall within the range of the expert assessments [18].
(TIF)

S5 Fig Calibrated parameter distributions for the uniformly-distributed interpretation of the expert assessment. Shaded areas represent the assumed wide prior probability distributions. Dashed red lines show distributions inferred by updating with the expert assessment. Solid black lines show posterior distributions from the combination of the expert assessment [18], paleoclimatic and instrumental observations, and the IPCC data [33].
(TIF)

S6 Fig Calibrated parameter distributions for the beta-distributed interpretation of the expert assessment. Shaded areas represent the assumed wide prior probability distributions. Dashed red lines show distributions inferred by updating with the expert assessment. Solid black lines show posterior distributions from the combination of the expert assessment [18], paleoclimatic and instrumental observations, and the IPCC data [33].
(TIF)

S7 Fig Calibrated parameter distributions for the normally-distributed interpretation of the expert assessment. Shaded areas represent the assumed wide prior probability distributions. Dashed red lines show distributions inferred by updating with the expert assessment. Solid black lines show posterior distributions from the combination of the expert assessment [18], paleoclimatic and instrumental observations, and the IPCC data [33].
(TIF)

S8 Fig Hindcasts from the inversions. Hindcasts of Antarctic ice sheet contribution to sea level from probabilistic inversion of expert assessments [18] (dashed red lines) and from combining expert assessments, paleoclimatic data, instrumental observations, and trends from the IPCC [33] using coupled probabilistic-Bayesian inversion (solid lines and shaded region). Shown are (a) uniform, (b) beta, and (c) normal interpretations of the expert assessment.
(TIF)

S1 Text Discussion of secondary mode in inferred expert priors. (PDF)
