# Supplementary figures and images for: Probabilistic inversion of expert assessments to inform projections about Antarctic ice sheet responses
Source: PLoS One. 2017 Dec 29;12(12):e0190115. doi: 10.1371/journal.pone.0190115 (PMC5747452; doi:10.1371/journal.pone.0190115)

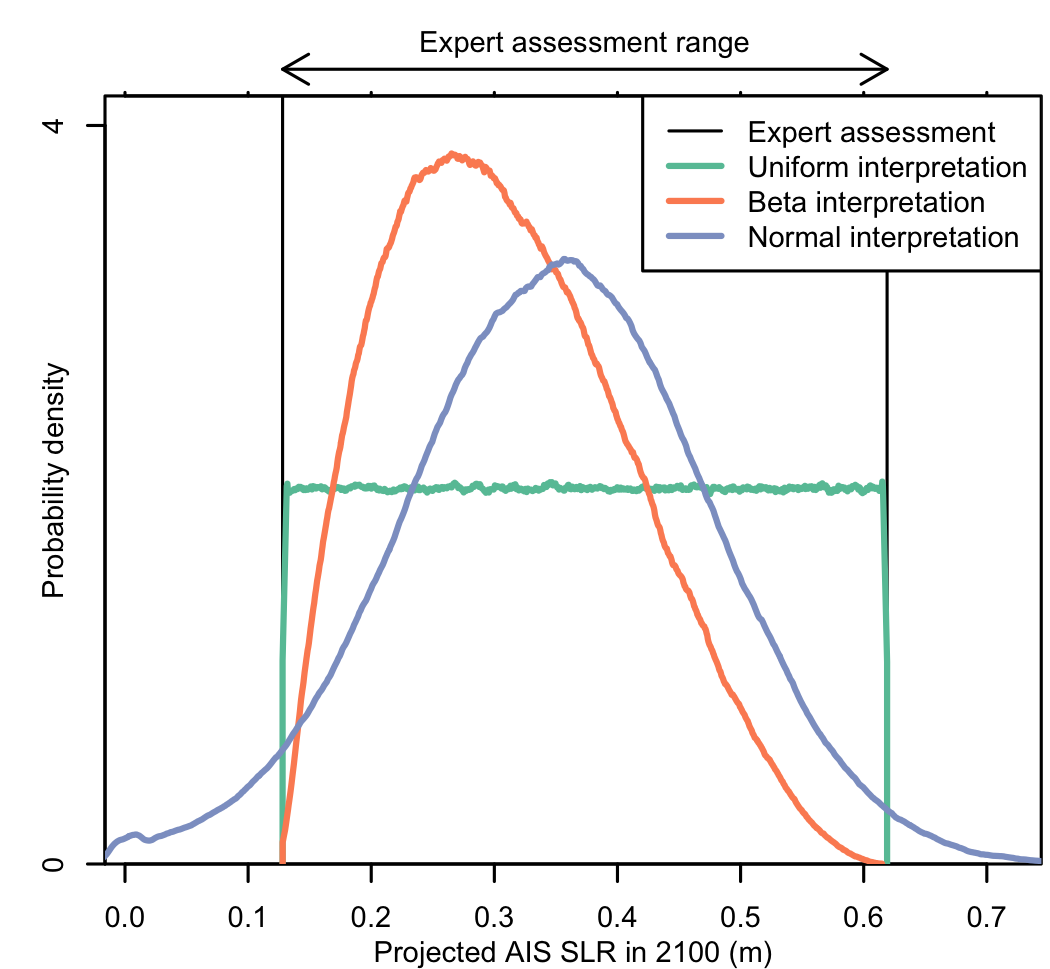

Supplement: S1 Fig — Lines give posterior probability distribution from probabilistic inversion of expert assessments. Vertical lines demarcate the range provided by Pfeffer et al. [18]. (TIF) [file pone.0190115.s001.tif]

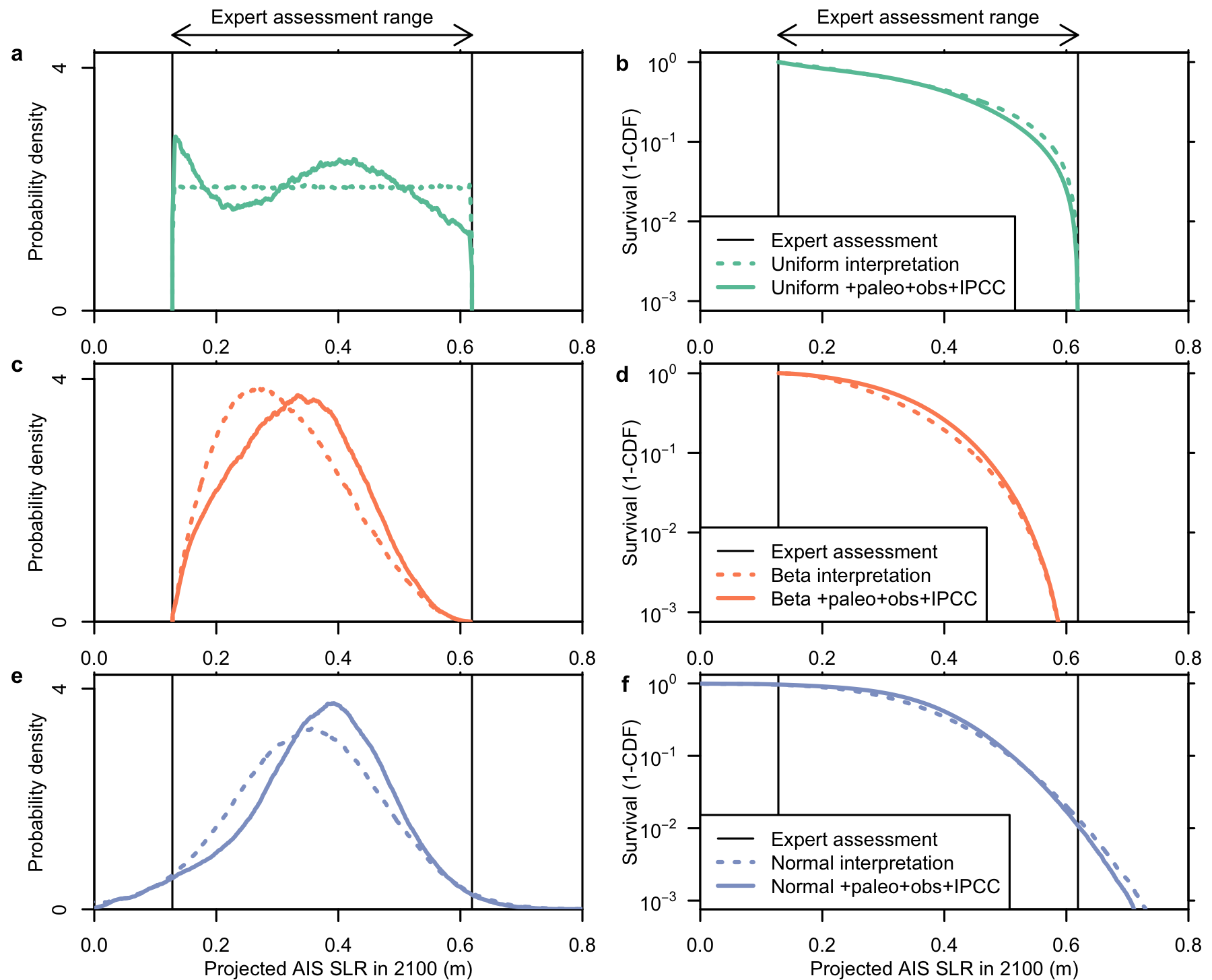

Supplement: S2 Fig — Dashed lines give sea-level estimates from probabilistic inversion of expert assessments. Solid lines give sea-level estimates from combining expert assessments, paleoclimatic data, instrumental observations, and modelled trends. Shown are (a-b) uniform, (c-d) beta, and (e-f) normal interpretations of the expert assessments. Vertical lines demarcate the range provided by Pfeffer et al. [18]. (TIF) [file pone.0190115.s002.tif]

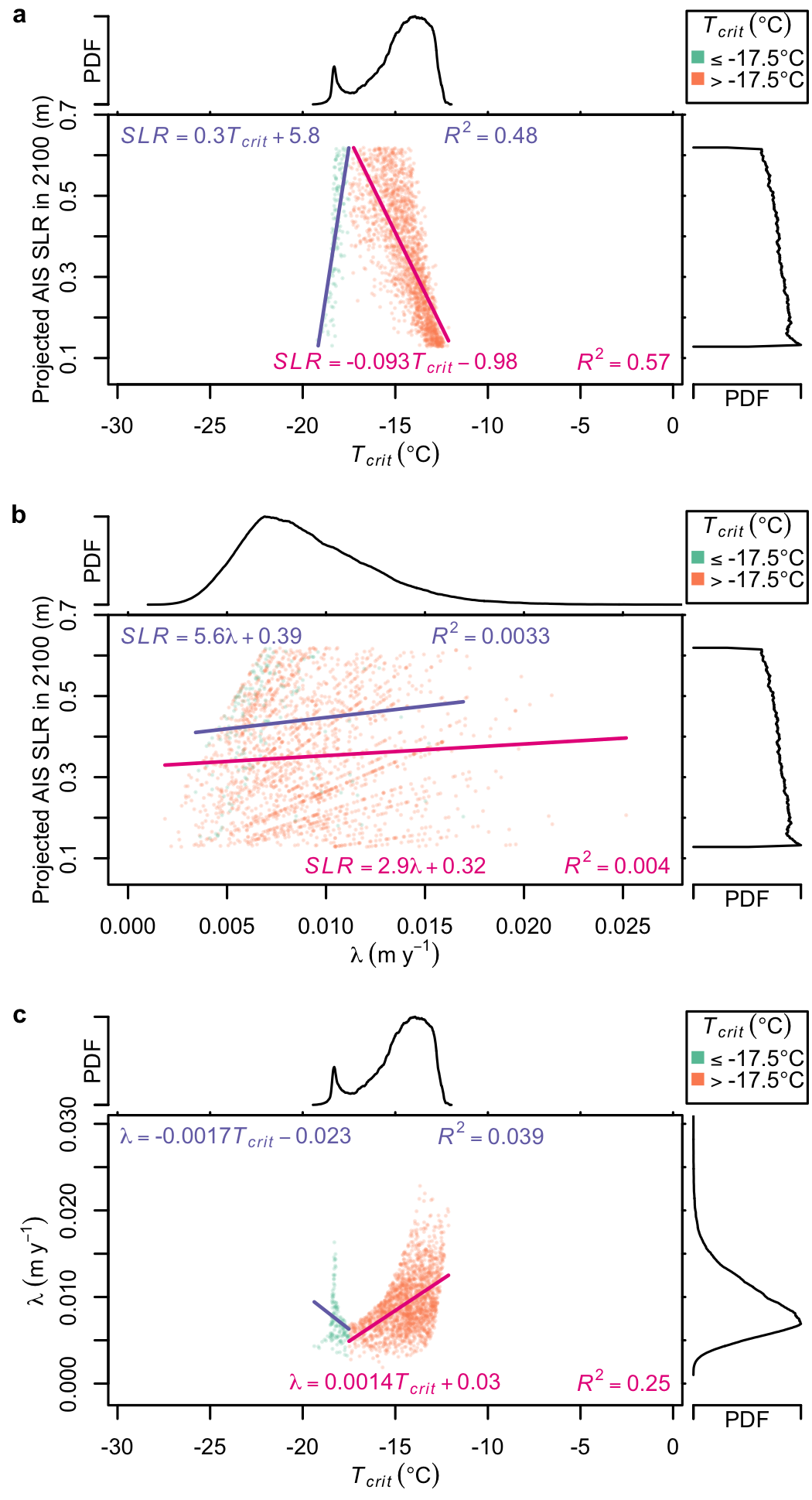

Supplement: S3 Fig — Shown are (a) covariance of Tcrit and sea-level estimates, (b) covariance of λ and sea-level estimates, and (c) marginal posterior distribution of fast dynamics parameters from probabilistic inversion of the uniform expert prior with all other parameters fixed at their joint maximum likelihood estimate. (TIF) [file pone.0190115.s003.tif]

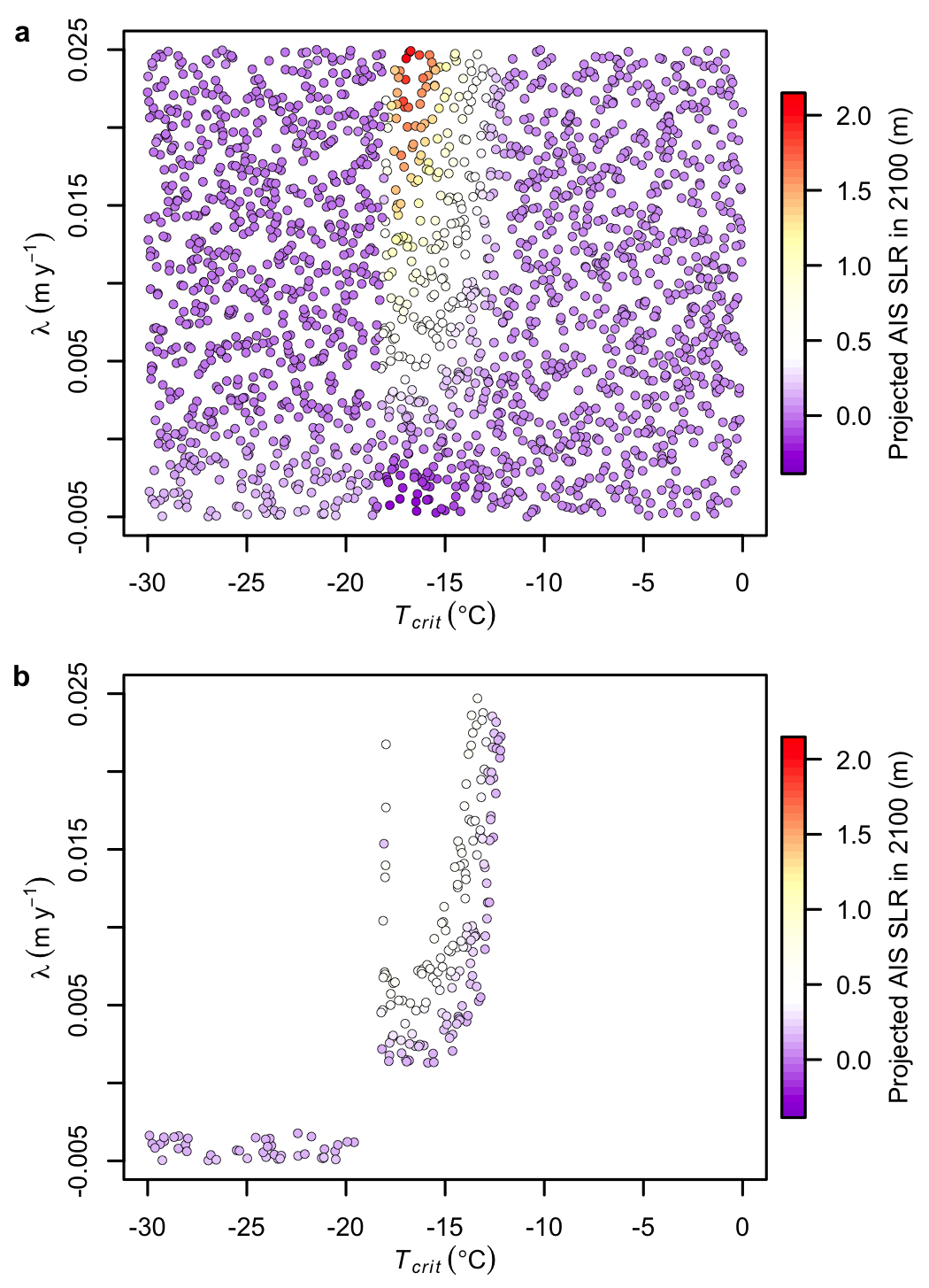

Supplement: S4 Fig — Shown are (a) all model runs and the (b) subset of those model runs that fall within the range of the expert assessments [18]. (TIF) [file pone.0190115.s004.tif]

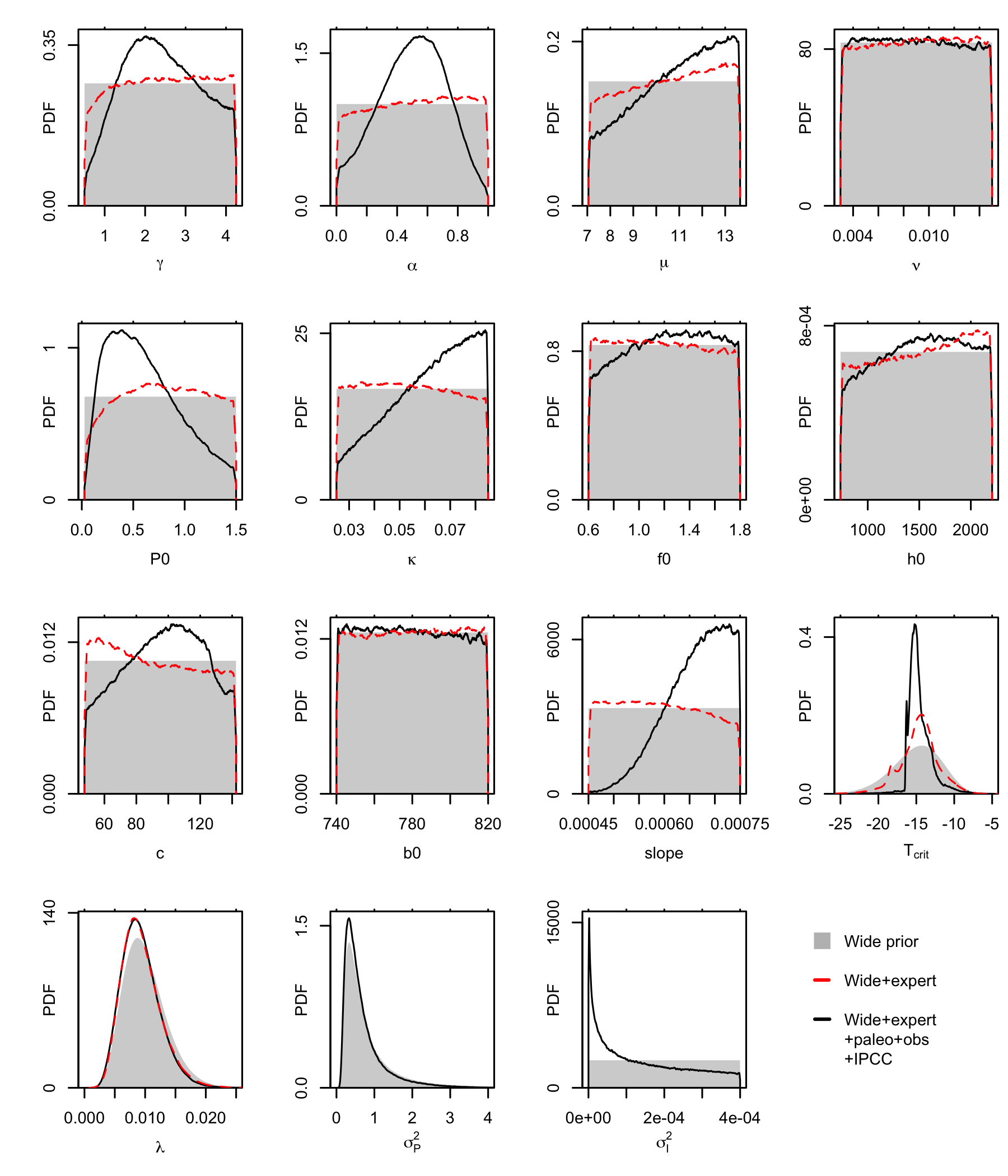

Supplement: S5 Fig — Shaded areas represent the assumed wide prior probability distributions. Dashed red lines show distributions inferred by updating with the expert assessment. Solid black lines show posterior distributions from the combination of the expert assessment [18], paleoclimatic and instrumental observations, and the IPCC data [33]. (TIF) [file pone.0190115.s005.tif]

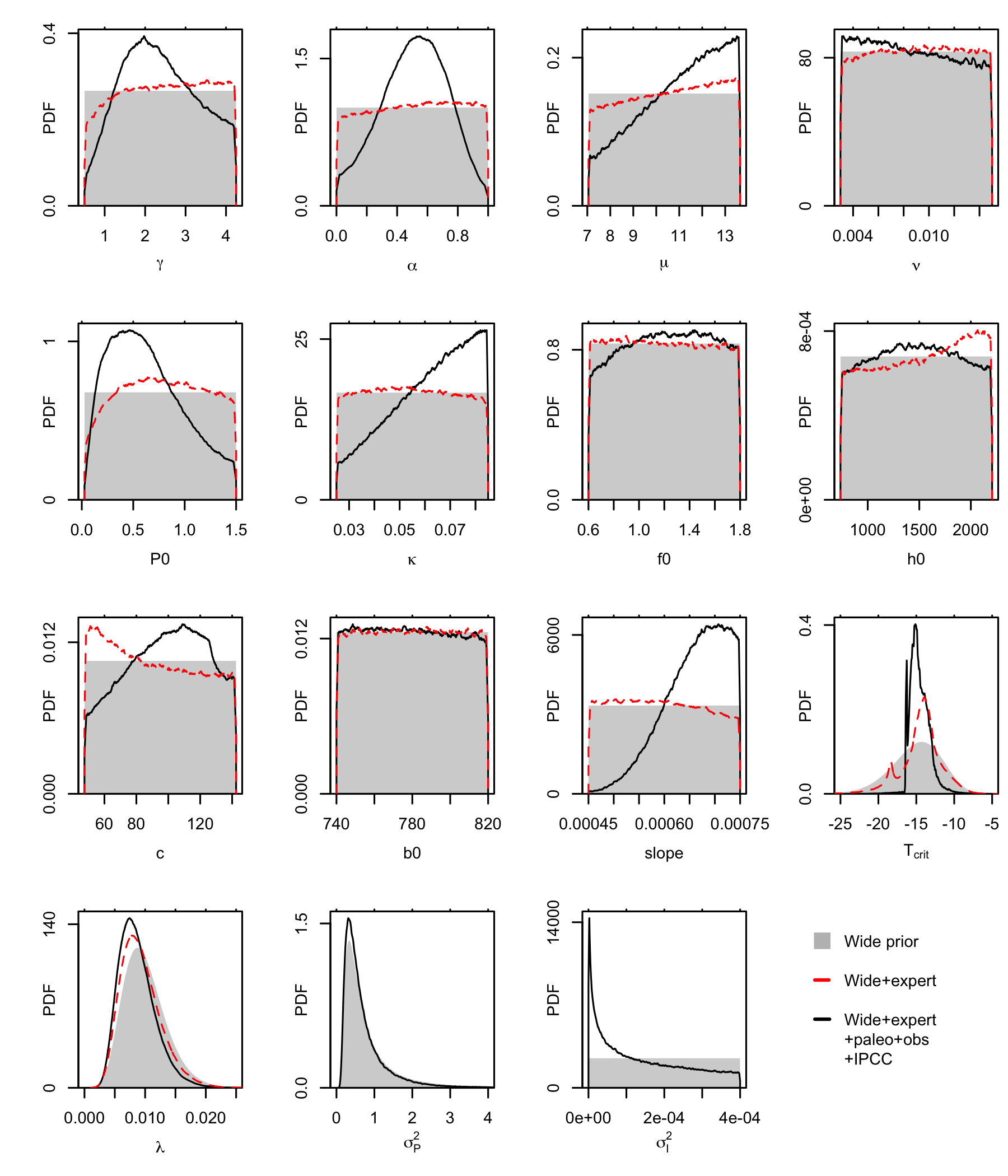

Supplement: S6 Fig — Shaded areas represent the assumed wide prior probability distributions. Dashed red lines show distributions inferred by updating with the expert assessment. Solid black lines show posterior distributions from the combination of the expert assessment [18], paleoclimatic and instrumental observations, and the IPCC data [33]. (TIF) [file pone.0190115.s006.tif]

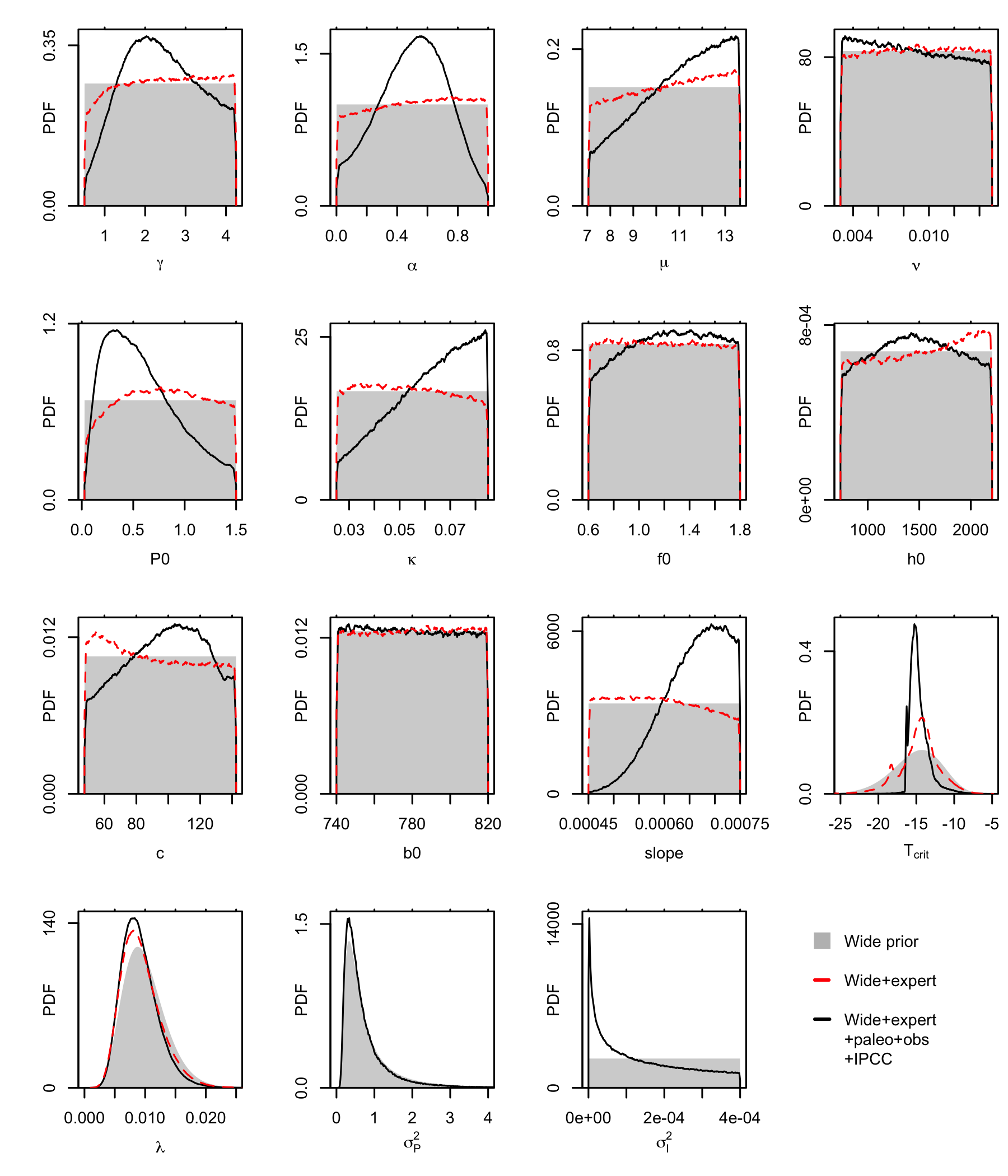

Supplement: S7 Fig — Shaded areas represent the assumed wide prior probability distributions. Dashed red lines show distributions inferred by updating with the expert assessment. Solid black lines show posterior distributions from the combination of the expert assessment [18], paleoclimatic and instrumental observations, and the IPCC data [33]. (TIF) [file pone.0190115.s007.tif]

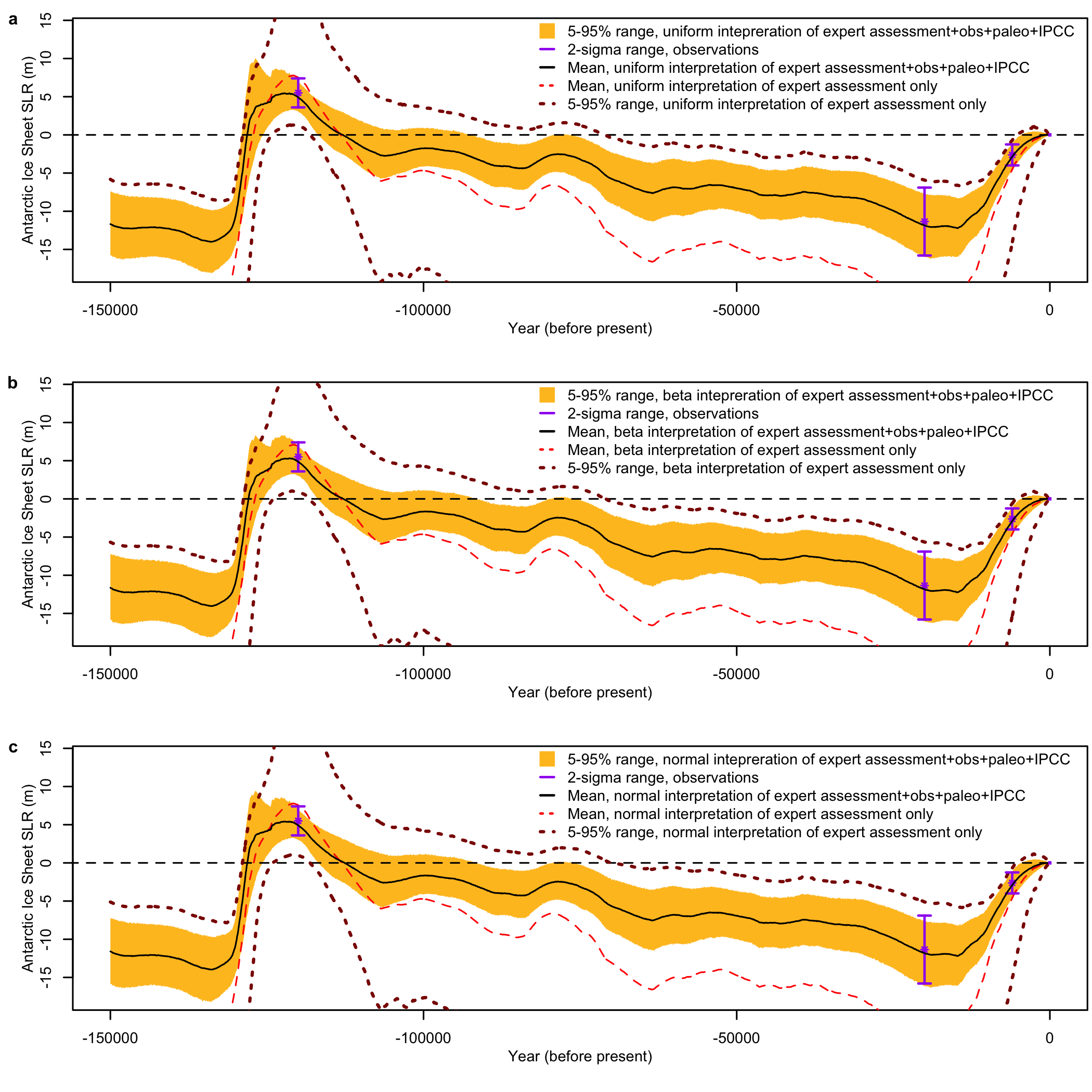

Supplement: S8 Fig — Hindcasts of Antarctic ice sheet contribution to sea level from probabilistic inversion of expert assessments [18] (dashed red lines) and from combining expert assessments, paleoclimatic data, instrumental observations, and trends from the IPCC [33] using coupled probabilistic-Bayesian inversion (solid lines and shaded region). Shown are (a) uniform, (b) beta, and (c) normal interpretations of the expert assessment. (TIF) [file pone.0190115.s008.tif]
