# Supplementary material for: Probabilistic inversion of expert assessments to inform projections about Antarctic ice sheet responses
Source: PLoS One. 2017 Dec 29;12(12):e0190115. doi: 10.1371/journal.pone.0190115 (PMC5747452; doi:10.1371/journal.pone.0190115)
Supplement: S1 Text — (PDF) [file pone.0190115.s009.pdf]

Supplementary material for:

Probabilistic inversion of expert assessments to inform projections about Antarctic Ice Sheet responses

Robert William Fuller[1], Tony E. Wong[2], and Klaus Keller[1, 2, 3] *

[1] Department of Geosciences, The Pennsylvania State University, University Park, Pennsylvania, United States of America

[2] Earth and Environmental Systems Institute, The Pennsylvania State University, University Park, Pennsylvania, United States of America

[3] Department of Engineering and Public Policy, Carnegie Mellon University, Pittsburgh, Pennsylvania, United States of America

* Corresponding author:

E-mail: klaus@psu.edu

Discussion of secondary mode in inferred expert priors

After the probabilistic inversion—where we use only the expert assessment to constrain the fast dynamics parameters (λ and T_{crit})—the inferred expert prior ranges relative to the wide prior ranges are displayed in Fig 2a. These differ from the parameter distributions that result from the hybrid inversion (Fig 2b), when we use the paleoclimatic and instrumental data to further constrain the parameters. We note that the Last Interglacial period (LIG) is the only time in the hindcast simulations during which the fast dynamic disintegration is potentially triggered. Thus, the additional constraint seen in Fig 2b relative to Fig 2a can be attributed to incorporating the LIG datum in the calibration period. The reduction of the secondary mode in the distribution for T_{crit} (Fig 2a versus Fig 2b) is a result of eliminating parameter sets which would trigger the fast dynamics too frequently (T_{crit} too low), thereby yielding simulations that overestimate the volume of disintegrated ice during the LIG. Similarly, we attribute the decreased upper range for

T_{crit} to eliminating parameter sets that would yield simulations that do not trigger the fast dynamics frequently enough, thereby underestimating the volume of disintegrated ice during the LIG. In the following text, we discuss and interpret this secondary mode when only the expert assessment is assimilated in the calibration.

We investigate the source of the secondary mode in T_{crit} (Fig 2a and S3c Fig) using supplemental experiments. First, we vary only the fast dynamics parameters, constrained only by the expert assessments. We hold the other model parameters fixed at a maximum posterior probability estimate from a probabilistic inversion of the expert assessments. The results demonstrate the mechanistic relationship between the fast dynamics parameters (S3 Fig). The results show that the rate of fast dynamical disintegration, λ, is inversely proportional to the timescale of the threshold response. Low values for λ equate to longer timescales, whereas high values equate to shorter timescales. Thus, as T_{crit} increases, more warming is required to trigger fast dynamics, and shorter timescales for disintegrating ice are required in order not to underestimate the expert assessments. Hence, as T_{crit} increases, λ increases, although there is an exceptional local maximum in λ at low values of T_{crit} (about -18.5 °C), which is explained later (S3c Fig). Perhaps counterintuitively, higher rates of disintegration do not imply higher sea-level estimates. In fact, sea-level estimates do not correlate to λ (S3b Fig). Taken together, Fig 2 and S3 Fig illustrate how threshold responses behave with respect to timescale.

Through this supplemental experiment, we find that sea-level estimates reach their maximum at intermediate values of T_{crit} (S3a Fig). At low values of T_{crit}, the probability of triggering fast dynamics is high, which requires a long response timescale in order not to overestimate the expert assessments. Hence, as T_{crit} increases from low to intermediate values, the timescale does not have to be as long, and sea-level estimates reach their maximum. As T_{crit} increases further from intermediate to high values, the probability of triggering fast dynamics decreases and sea-level estimates decrease.

In a second experiment, we explore the parameter space of the fast dynamics parameters using Latin hypercube sampling [1]. We further widen the assumed priors for the fast dynamics parameters, as shown by the range of the figure (S4 Fig). We hold the other model parameters

fixed at a maximum likelihood estimate. We use Latin hypercube sampling to generate the fixed parameters, with their likelihood constrained by all of the data, including the expert assessments and the paleoclimatic windows. We constrain the fast dynamics parameters using the expert assessments, but not the paleoclimatic calibration windows (S4b Fig).

The results show that the model simulations with a low trigger temperature, T_{crit}, and a high disintegration rate, λ, still agree with the expert assessments (S4b Fig). This is because fast dynamics are no longer triggered once the volume of the ice sheet falls below 18 million km^3. This prevents anomalous combinations of fast dynamics parameters from melting the entire ice sheet during the Last Interglacial (LIG), which is not included as a constraint in the Latin hypercube sampling or the probabilistic inversion. Adding the LIG as a constraint in the coupled probabilistic-Bayesian inversion eliminates the secondary mode (Fig 2b).

The Latin hypercube sampling of the widened fast dynamics parameters (S4 Fig) further shows that the model does not necessarily reject non-physical values for the fast dynamics parameters (i.e. model runs where the disintegration rate, λ, is less than zero or T_{crit} is so low that all of the susceptible ice would have melted long ago). We further find that the expert assessments create a discontinuity in parameter space between non-physical and more physically plausible fast dynamics parameters (S4b Fig). Initializing the random-walk Metropolis-Hastings algorithm with non-physical values for the fast dynamics parameters would make it much more difficult to sample physically plausible values.

References

1. McKay MD, Beckman RJ, Conover WJ. A comparison of three methods for selecting values of input variables in the analysis of output from a computer code. Technometrics. 1979;21: 239. doi:10.2307/1268522
